# Advances in Imaging Modalities, Artificial Intelligence, and Single Cell Biomarker Analysis, and Their Applications in Cytopathology

**DOI:** 10.3389/fmed.2021.689954

**Published:** 2021-07-02

**Authors:** Ryan P. Lau, Teresa H. Kim, Jianyu Rao

**Affiliations:** Department of Pathology and Laboratory Medicine, David Geffen School of Medicine at the University of California, Los Angeles, CA, United States

**Keywords:** single cell biomarker analysis, computational cytopathology, multiplex immunofluorescence, molecular cytopathology, computational pathology

## Abstract

Several advances in recent decades in digital imaging, artificial intelligence, and multiplex modalities have improved our ability to automatically analyze and interpret imaging data. Imaging technologies such as optical coherence tomography, optical projection tomography, and quantitative phase microscopy allow analysis of tissues and cells in 3-dimensions and with subcellular granularity. Improvements in computer vision and machine learning have made algorithms more successful in automatically identifying important features to diagnose disease. Many new automated multiplex modalities such as antibody barcoding with cleavable DNA (ABCD), single cell analysis for tumor phenotyping (SCANT), fast analytical screening technique fine needle aspiration (FAST-FNA), and portable fluorescence-based image cytometry analyzer (CytoPAN) are under investigation. These have shown great promise in their ability to automatically analyze several biomarkers concurrently with high sensitivity, even in paucicellular samples, lending themselves well as tools in FNA. Not yet widely adopted for clinical use, many have successfully been applied to human samples. Once clinically validated, some of these technologies are poised to change the routine practice of cytopathology.

## Introduction

With advancements in computer vision and machine learning, artificial intelligence (AI) and deep learning algorithms have made huge strides in their ability to analyze and interpret imaging data. Cytology was the first setting in which AI was implemented in the pathology laboratories, in the form of computer-assisted Pap test screening. Initially developed with the intention of diagnosing rather than screening, there was significant debate about their broad application to clinical samples. These early systems used morphometric differences to distinguish normal from abnormal cell populations, but due to morphometric overlap between cell populations, and artifact from conventional cytology specimen preparation such as air drying and obscuring blood, the frequently encountered inaccuracies made these tools better suited for screening tests (1). Since then, image analysis has been applied predominantly to surgical pathology or histopathology samples. With recent improvements in imaging capture technologies and digital image analysis, the desire for computational cytopathology tools is growing. Though advances in computational tools to aid in morphometric diagnosis is an important initial step, in our ever complicated time of precision medicine this alone remains insufficient. Predictive and prognostic ancillary testing is becoming increasingly important, and more and more information is expected to be ascertained from smaller and smaller tissue biopsies or aspirated samples (2). In this review we will highlight some advances in imaging capabilities that have unique potential applications to cytology specimens, including exfoliated samples (such as cervical samples, urine and body cavity fluid samples), aspirated samples (fine needle aspiration, FNA), or liquid biopsy samples (circulating tumor cells, CTC). We will review the current state of deep machine learning and AI and how these algorithms can aid in cytologic diagnosis, and then focus on recent multiplex systems that can analyze biomarkers in small, sometimes single cell samples. The intersection of novel multiplex tools and automated imaging and analysis is still in investigational stages, but has shown very promising proof of concept and may soon become part of routine clinical medicine.

## New Imaging Modalities for Three-Dimensional Analysis

An oft-cited challenge in cytology image processing is the three-dimensional (3D) nature of a cytology specimen compared to a histology slide, and early inaccuracies in Pap test computer-assisted screening were partially attributed to the cellular overlap in conventional cervical cytology smears (1). However, recent developments have allowed the acquisition of high-resolution three-dimensional images described below, as reviewed by Pantanowitz et al. (3). Though still underutilized and under-examined insofar as it pertains to the field of cytopathology, these technologies have shown promise in assessing tissues in three dimensions with a previously unattainable level of granularity that is sure to have relevant applications.

Optical coherence tomography (OCT) technology uses light and measurement of back-reflected light, conceptually similar to ultrasound, to examine tissues at the subcellular level. Studies have shown the ability of OCT technology to distinguish normal from neoplastic tissue, though it has been adopted more widely in clinical fields for *in situ* imaging than in anatomic pathology. Inasmuch as it pertains to the field of gynecologic pathology and by proxy, cervical cytopathology, a study reported an 88% sensitivity and 69% specificity in detecting high-grade squamous epithelial lesions in loop electrosurgical excision procedure specimens (4). Other cytologic settings where this technology has been applied involve identification of abnormalities on endoscopic ultrasound-guided fine needle aspiration specimens and pelvic washings. While this technology showed promise with identifying malignant tumor clusters, its efficacy in assessing nuclear details and single cell abnormalities such as gastric signet ring cell carcinoma was less pronounced (5, 6).

Novel imaging techniques also exist that allow 3D imaging of cells in suspension. The Cell-CT instrument employs optical projection tomography and uses a scanning objective lens that can assess single cells and bare nuclei suspended in a microcapillary tube. Similar to a computed tomography (CT) scan, the instrument takes 500 two-dimensional (2D) images of individual cells, and a 3D reconstructed image is produced, with reported resolution close to 1 micrometer, allowing for analysis of sub-cellular features that might not be apparent in routine 2D analysis (7). With the collection of large libraries of 3D reconstructions, algorithms can be trained to identify abnormal cells that can be reviewed by cytologists and cytopathologists.

Another novel microscopy technique is quantitative phase microscopy that relies on light interference effect to identify cellular features at a resolution near 1 nanometer. The refractive index of cell nuclei as measured by quantitative phase microscopy has been shown to be a sensitive measure of malignancy or premalignancy, and can identify subtle aberrations that were not identified by routine histopathologic examination in breast cancer specimens (8). Since that early study, this imaging technique has identified atypical or malignant cells in numerous settings, including bile duct biopsies, colon biopsies, cervical cytology specimens, and urine cytology (3).

These novel microscopy and image capturing techniques have shown promise in their ability to analyze diseased samples at the cellular and subcellular level. Further studies will undoubtedly show their use in more routine clinical practice. Their development and adoption may diminish many of the unique obstacles that have heretofore favored the application of image analysis and computational pathology to histopathology over cytopathology.

## Artificial Intelligence in Cell-Based Image Analysis

Concurrent with the advances in image capture technology has been an increased demand for image analysis. In recent years, the interest in developing computational methods to screen, diagnose, and streamline pathology workflow has increased dramatically. Advances in AI, image analysis, and deep learning are augmenting the myriad ways that computational pathology can be applied to cytopathology.

Machine learning is the branch of AI that pertains to the ability of computer algorithms to improve and perform tasks without explicit instruction. It is the use of algorithms to analyze data, automatically learn, and then apply that to new data to make intelligent decisions. Conventional machine learning requires significant engineering and manual input. Based on linear techniques, it has limited ability to process raw data, and this can only be performed with a predetermined set of instructions. With the historical use of conventional machine learning, these algorithms were much less effective when encountering new variables that had not been programmed. Deep machine learning is a more novel subfield of machine learning that employs artificial neural networks (ANN). Inspired by the nervous system, these ANN are complex models with interconnected “neurons” structured into a hierarchy of multiple layers. It is inherently non-linear and too complex to be programmed manually, eliminating the need for manual input of labels to handle large sets of data. With deep machine learning, feature extraction is an inherent part of the computer program rather than determined by human input (9, 10). In contrast to conventional machine learning, these algorithms utilize multiple levels of abstraction that allows the algorithm to adjust and adapt on its own to newly introduced variables. Deep learning is particularly useful with very large and unstructured data sets, and excels with complex problems such as image classification. Its applications to the medical sciences are being increasingly explored (11, 12).

One of the earliest successes of deep learning was image classification, or the ability to give a meaningful label to an image. Initially trained through large labeled image libraries, image classification programs are an example of supervised learning wherein the end goal is to classify something as “x” or “not x.” Different features or explanatory variables are then weighted based on the data gathered from the training set. Through the use of deep learning to extract feature vectors, image classification models have become even more granular in their ability to identify differentiating features in cell morphology (13, 14). Furthermore, the application of image segmentation—identifying unique components of an image by partitioning an image into multiple components or pixels—has also increased the granularity at which image analysis can be performed. For example, assigning a label to every pixel in an image allows grouping of pixels that share certain characteristics; this can enable an algorithm to examine features of an individual cell nucleus independent of the entire cellular image (13).

## Single Cell Biomarker Analysis

The current era of medicine can seem full of contradictions. With advances in molecular diagnostics and targeted therapy, more and more predictive and prognostic ancillary tests are being performed. Simultaneously, ever-burgeoning healthcare costs have highlighted the need for proper resource utilization (15). It is in this context where precision medicine and resource utilization can sometimes seem at odds, and the molecular cytopathologist is tasked with the role of tissue custodian (16).

Precision medicine, guided by predictive biomarkers of individual patients' tumors, has increased overall survival as well as reduced treatment-associated toxicity (17). Assessment of several biomarkers is routinely recommended before administration of therapy. The “gold standard” remains tissue biopsy diagnosis - particularly for clinical trials. However, in routine clinical practice many patients are diagnosed on small endoscopic biopsies and cytologic materials. Repeated studies have also demonstrated higher quality of nucleic acids in cytologic preparations compared to histologic samples (18, 19). Despite the validation of cytologic preparations for ancillary biomarker testing, cytology faces unique challenges including the discohesive nature of the samples, mixed cellular populations, cell exhaustion, and inconsistent localization of cells between levels in cell block preparations. These obstacles necessitate a somewhat more gestalt assessment of biomarkers that can contribute to higher inter-observer variability (20–22). However, cell based analysis, unlike tissue section analysis, has an important advantage as it typically measures the biomarker expression in a whole cell, rather than a section of a cell, which conceivably is more accurate for quantitative assessment of the expression of a specific biomarker.

## Multiplex Assay in Conjunction With Single Cell Analysis

Multiplex immunohistochemistry/immunofluorescence (M-IHC/IF) allows *in situ* visualization of multiple markers, and thus cell types, in the same slide or specimen. This ability to concurrently examine different populations of cells *in situ* adds a level of refinement that opens up doors to explore the tumor microenvironment.

M-IHC/IF assays have shown great promise in assessing biomarkers. Quantitative fluorescence image analysis is an established technology that allows the evaluation of multiple biomarkers, *in situ*, in single cells. When used as an adjunct to conventional cytomorphology, it can increase the specificity and sensitivity of tumor detection (23, 24). Several studies have also examined proximity of PD-1 and PD-L1, CD8+ cell density, and T-cell activation, and shown an improved ability to predict tumor response to anti PD-1/PD-L1 therapy (25). Specific to cytology, M-IF has been applied to pleural effusions with the ability to identify and quantify individual cell types (26).

Concurrent advances in digital imaging have also presented the ability for virtual multiplex assays. Using image reconstruction from scanned whole slide images, individual levels can be more accurately layered with computer software. This has demonstrated an increased ability for the observer to identify antibody co-expression as well as filter out contaminating cell populations (20).

Some limitations of standard multiplexed technologies are a finite amount of markers due to spectral overlap, sample loss, inability to perform concurrent genetic and proteomic analysis on single cells, and long cycle times. Though not yet a routine part of clinical use, several exciting multiplexing technologies are in development with the ability to assess an expanded panel of proteins.

**Figure 1:**
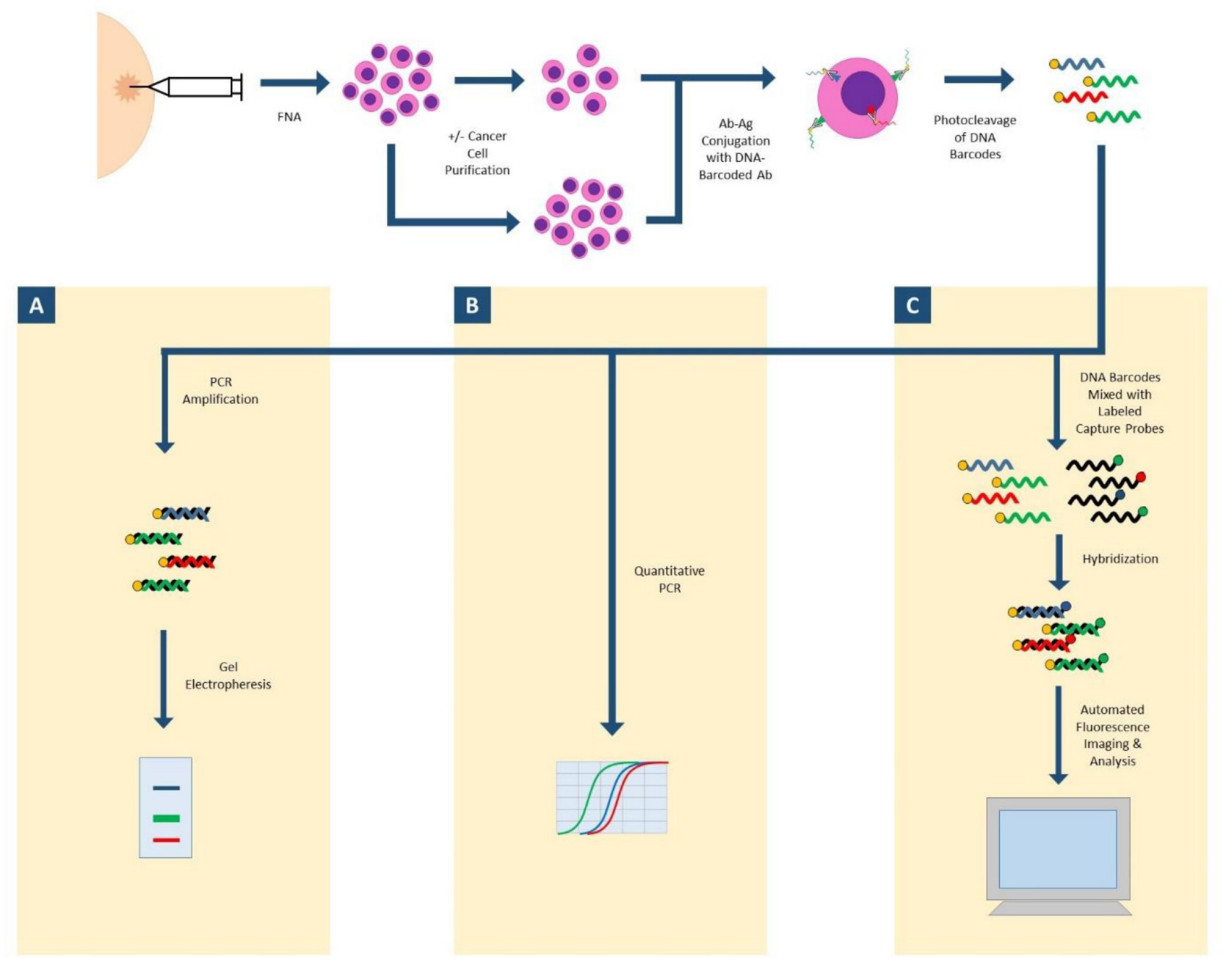
ABCD (antibody barcoding with cleavable DNA). FNA biopsy is performed to isolate cells of interest, which may undergo a purification step. Cells are incubated with DNA-barcoded antibodies to proteins of interest. DNA barcodes are cleaved from antibodies. **(A)** Released DNA-barcodes can undergo PCR amplification and gel electrophoresis for semi-quantitative measurement of protein expression; **(B)** Released DNA-barcodes can undergo multiplex quantitative PCR; **(C)** Released DNA-barcodes can be hybridized with fluorescent labeled capture probes and automatically imaged and analyzed with nanostring technology.

Below are some examples of single cell based multiplex analysis technologies:

**Antibody Barcoding with cleavable DNA (ABCD)** is a technology that can assess hundreds of proteins in small cellular samples, such as FNA (Figure 1). This method uses unique DNA “barcodes” to tag each antibody of interest. Then, using proteolytic cleavage and photocleavage, the DNA barcode is released from the antibody and protein of interest. Released barcodes can be quantitatively or semi-quantitatively assessed with a number of different modalities. DNA barcodes can undergo polymerase chain reaction (PCR) with gel electrophoresis to semiquantitatively assess protein expression. DNA barcodes can also undergo quantitative PCR to analyze protein expression. Most recently, nanostring technology was successfully applied to released DNA barcodes in human FNA samples. The released DNA barcodes are hybridized to a chain of fluorescent tags, which are optically detected by a computer. This highly sensitive assay requires no amplification step and the entire process is completed in hours. In proof-of-concept studies, up to 90 individual proteins were able to be analyzed, and results in even single cell profiles had correlations as high as 0.96 when compared to larger bulk samples (27, 28).

**Figure 2:**
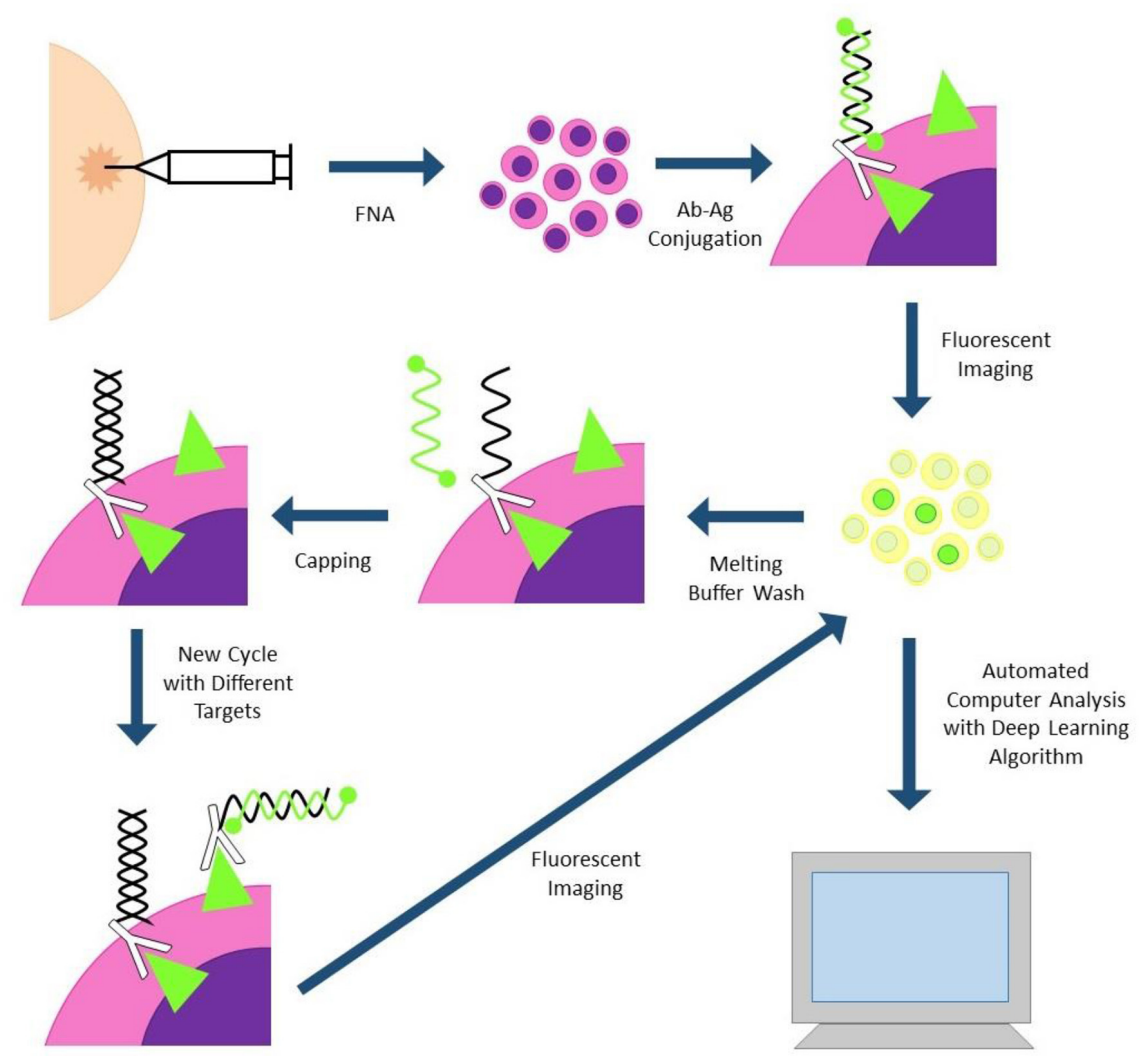
SCANT (single cell analysis for tumor phenotyping). Cells are obtained via fine needle aspiration biopsy and incubated with DNA-antibody conjugates. DNA strands are hybridized to complementary strands with two flurochromes; antibodies with different flurochromes will fluoresce at different channels. Cells are imaged and subjected to automated image analysis. Fluorescent strands can be washed off and capped between cycles to reduce cycle-to-cycle background.

**Single Cell Analysis for Tumor Phenotyping (SCANT)** technology has also been successfully applied to clinical samples (Figure 2). This technology uses DNA-barcoded antibodies to simultaneously analyze expression of multiple proteins. Cells are incubated with DNA-barcoded antibodies to proteins of interest, then fluorochrome tagged complementary nucleic acid strands are conjugated to each unique DNA barcode. Differentially tagged complementary strands fluoresce at different channels, allowing for assessment of multiple proteins simultaneously. Fluorescent images can be automatically analyzed by computer program and deep learning algorithms. Fluorescent tagged complementary strands can also be washed off between cycles, decreasing the amount of background signal between cycles. One drawback to this technology is the long destaining time between cycles which can take several hours for the sample to be fully processed (29).

**Figure 3:**
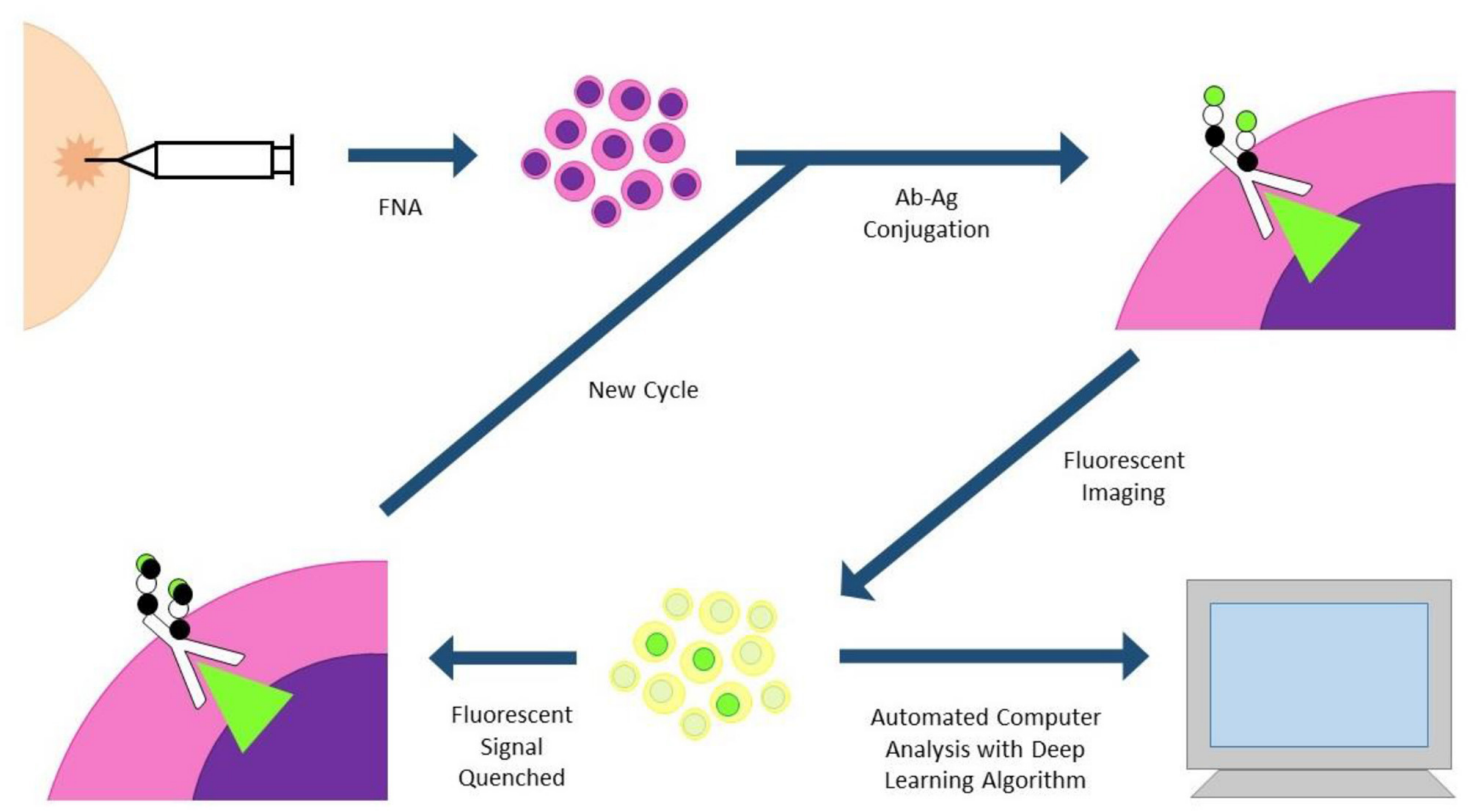
FAST-FNA (fast analytical screening technique fine needle aspiration). FNA biopsy samples are fixed and stained with fluorescent-labeled antibodies. Antibodies are conjugated to trans-cyclooctene (TCO), conjugated to tetrazine (Tz), conjugated to a fluorescent label. Images are processed in an automated image cytometer; deep learning algorithms quantify marker expression. Tz-activated black hole quenchers quench the fluorescent signal in mere seconds, allowing much shorter time intervals between cycles.

**Fast Analytical Screening Technique FNA (FAST-FNA)** fixes samples with barcoded antibodies in a cyclic fashion that are then imaged with an automated image cytometer (Figure 3). Antibodies are conjugated to trans-cyclooctene, conjugated to tetrazine, conjugated to a fluorescent label. After antibody-antigen conjugation, the cells undergo fluorescent imaging, and images are automatically analyzed by computer programs and deep learning algorithms. After image acquisition, ultrafast destaining can be performed through a “click-chemistry” reaction, where a black hole quencher is activated by the tetrazine bound to the antibody, quenching the fluorescent signal in mere seconds. This significantly decreases the cycling time and signal-to-noise ratio that plagues other cycling technologies (30, 31). This methodology has already been applied to head and neck specimens, and two scores based on this new technology (FAST PD-L1, FAST cold/hot score) could become a more nuanced predictor of anti PD-1/PD-L1 therapy than standard combined positive score (CPS) currently used with PD-L1 immunohistochemistry (30, 32).

**Figure 4:**
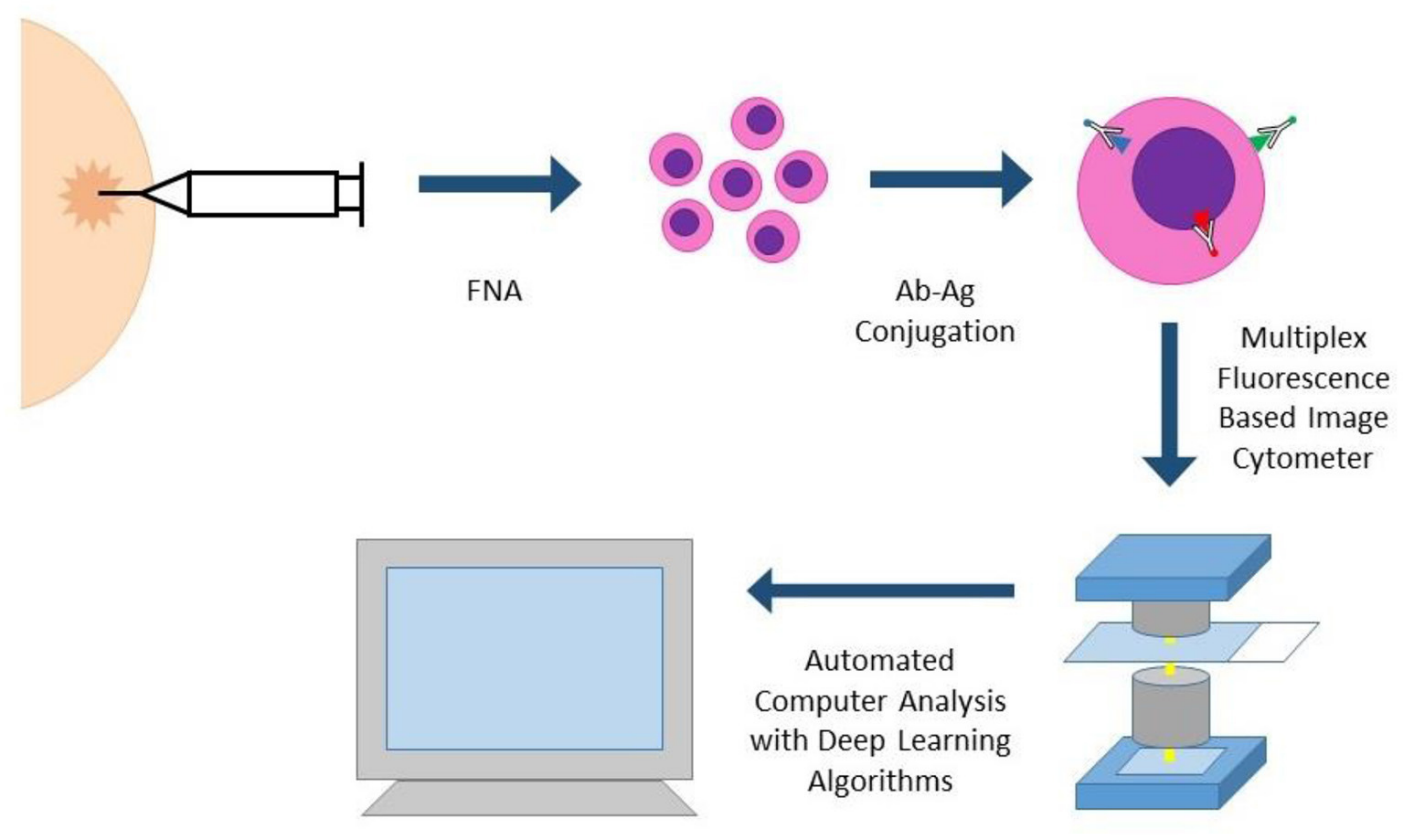
CYTO-PAN (portable fluorescence-based image cytometry analyser). This specific system was used to diagnose and subtype breast cancer in FNA specimens. FNA biopsy samples are processed with prefabricated kits with preselected antibodies. Slides with stained cells are analyzed by the CytoPAN device, a fluorescence based image cytometer with five optical channels. Custom-developed algorithms identify cancer cells and biomarker information, producing quantitative reports. The entire process could be performed within 1 h.

**Portable Fluorescence-based Image Cytometry Analyzer (CytoPAN)** is another exciting technology at the intersection of multiplex biomarker analysis and computational pathology (Figure 4). Using prefabricated kits with preselected, fluorescently labeled antibodies, the CytoPAN system employs multiplex immunostaining on FNA samples, which are then mounted in a glass substrate, imaged by the device, and run through an automated algorithm, requiring no user input. The CytoPAN system obtained diagnostic accuracy of 100% in diagnosing breast cancer, and diagnostic accuracy >90% for tumor subtyping of ER/PR and HER2 expressing tumors. Not only was it diagnostically successful, it has the added benefit of affordable cost per kit, 1 h turnaround time, and reliability even in paucicellular specimens (33).

## Applications of Cell-Based AI Analysis in Human Specimens

The application of these emerging tools has shown much promise in the fields of cytopathology, with successful data being published across organ systems. Several fantastic reviews exist, and the following are just a small sampling of the successful applications of these emerging technologies in human samples (32, 34).

### Gynecologic

In cervical cytopathology, cells may often cluster or overlap, producing artifact that is difficult for machine learning algorithms to distinguish. Many studies have been published using deep learning algorithms to attempt to image segmentation to analyze nuclei and cytoplasm separately. Using three main stages of cell detection, cytoplasm segmentation, and boundary refinement, recently published findings of a deep convoluted neural network based method has also shown superior performance in segmenting overlapping cells in cervical cytology specimens, addressing 3D layering, one of the common pitfalls in cytopathology (35).

### Urine

Artificial intelligence algorithms have a long history in urine cytology, and several have demonstrated superior results to human review (36–38). A recent study used a trained algorithm to successfully analyze whole slide images of urine cytology cases, with a sensitivity of 79.5% and specificity of 84.5% in detecting high grade urothelial carcinoma. Furthermore, it did it with astonishing speed, analyzing ~36,000 cells per case in under 8 min (39).

### Lung

The aforementioned OCT technology has been applied to sputum samples to assess for lung cancer. Using 3D morphologic analysis to select abnormal cells for subsequent manual review, there were reported sensitivities and specificities >90% in detecting lung cancer (40, 41). Deep machine learning has been applied to histopathology specimens in the lungs and shown success in classifying lung adenocarcinoma, lung squamous cell carcinoma, and benign lung. Furthermore, this deep convolutional neural network was able to predict common mutations found in lung adenocarcinoma (12).

### Breast

Advances in image segmentation has shown promise when applied to breast cancer; one study showed the ability of deep-learning-based image segmentation to identify cell types and cell states, allowing for a quantitative analysis of the tumor microenvironment in breast cancer samples (42). The novel multiplex technologies CytoPAN and SCANT have shown proof of concept in cancer detection, tumor subtyping, and interrogation of drug-relevant pathways in breast cancer (29, 33).

### Head and Neck

Computational pathology has been successfully applied to head and neck lesions. In the thyroid, repeated studies have examined nuclear parameters and shown success in distinguishing benign from malignant lesions. However, most of these studies proved successful only in analyzing binary outcomes: malignant or benign. Many of these have been small studies and have not demonstrated enough strength to be applied to routine clinical work (32, 43, 44). One study has shown success in distinguishing benign from malignant tumors in the parotid gland (45). In the head and neck, the overall utility of digital image analysis remains unknown as its application to indeterminate categories—those cases that are clinically most in need of diagnostic refinement—has not been studied or shown less success.

## Discussion

Advances have been made in recent decades in many exciting fields: image capture, data analysis and AI, and single cell biomarker testing. Each of these carry exciting promise to modify how the field of cytopathology practices. It is also at the intersection of all these emerging technologies that supremely nuanced information can be learned about individual patients' tumors. These technologies have the capability to streamline workflow, decrease turnaround time for diagnostic tests to mere hours, identify granular prognostic and predictive features to guide precision medicine, and cut costs and utilize resources more efficiently. Although they have shown significant promise in proof-of-concept studies, there are still many technical and cultural obstacles that must be overcome before they can be applied to routine clinical use.

Technically speaking, successful implementation of computational pathology and AI require huge amounts of data with large training sets. Large curated datasets must be consistently annotated and should be open-access for more robust performance. In regards to diagnosis in cytology, the computational tools that exist still primarily focus on image analysis; a host of other features such as past medical history, family history, genetic and socioeconomic factors, laboratory especially molecular studies, and radiology studies play a very important role in diagnosis and clinical decision making—all of which should be incorporated into algorithms. The vast computational resources needed for data analysis and storage also remain an impediment to wide adoption.

There are additional barriers to digitizing cytology vs. histologic samples due to the nature of the sample preparation. Cytologic preparation often uses whole cells smeared across a slide or made with a liquid-based preparation, causing 3-D overlap. The cytologic preparation requires evaluation of multiple z-planes, which takes more time to scan and review by a diagnostician (46, 47). Proper validation studies and training will likely alleviate the pressures against adopting these digital systems for routine use in cytology.

Furthermore, the successful implementation of digital pathology systems and AI depends on efficient regulation, which is currently nascent. Food and Drug Administration (FDA) approval is not required to use digital pathology systems in a clinical setting. Instead, they are designed, manufactured, and validated in individual laboratories as laboratory developed tests, and the College of American Pathologists (CAP) recommends explicit documentation of this in the pathology report. CAP and Clinical Laboratory Improvement Amendments (CLIA) also require a similar validation process for the use of AI-based technologies on patient samples (48). The development of a regulatory guidance for the use of machine learning technology is only in its early stages. The FDA has released proposals regarding the process of using machine learning software as a medical device, but has not specifically outlined its performance requirements as done for other CLIA-mandated laboratory tests (49). Therefore, a more complete regulatory framework is necessary before moving forward in the digital landscape.

Culturally, there may also exist reluctance to adopt these new technologies. A valid criticism of AI is that deep machine learning, relying on deep neural networks with multiple, non-linear, abstract layers, is inherently a “black box.” There can be a tendency in these algorithms for bias and/or discrimination, and the lack of transparency can make adjustments difficult. This underscores the need for explainable and transparent systems, ones in which developers can interrogate the algorithms and identify which features are weighted in decision making processes (50–52). The wide-scale adoption of deep learning into clinical medicine requires a certain degree of trust, which is dependent on a consistent contextual analysis of the problem, ongoing relationships with stakeholders, and respect of professional discretion (51).

Ultimately, with continued improvement in these modalities to accurately diagnose disease, further validation through studies with human subjects, approval by regulatory boards, and growing trust between stakeholders and these systems, these advances are poised to change the practice of cytopathology.

## Author Contributions

RL reviewed and synthesized the relevant information and was the primary author of the review article. TK reviewed and synthesized the relevant information and contributed figures and discussion of the review article. JR made substantial contributions to the conception, drafting, and revision of the article. All authors contributed to the article and approved the submitted version.

## Conflict of Interest

The authors declare that the research was conducted in the absence of any commercial or financial relationships that could be construed as a potential conflict of interest.
